# Symbiotic Nitrogen Fixation in Legume Nodules: Metabolism and Regulatory Mechanisms

**DOI:** 10.3390/ijms151119389

**Published:** 2014-10-24

**Authors:** Saad Sulieman, Lam-Son Phan Tran

**Affiliations:** 1 Signaling Pathway Research Unit, RIKEN Center for Sustainable Resource Science (CSRS), 1-7-22, Suehiro-cho, Tsurumi, Yokohama 230-0045, Japan; E-Mail: s.sulieman@psc.riken.jp; 2 Department of Agronomy, Faculty of Agriculture, University of Khartoum, Shambat, Khartoum North 13314, Sudan

**Keywords:** nitrogen fixation, legume, symbiosis, nitrogenase, nodule, carbon metabolism, nitrogen metabolism, oxygen supply, translocation

## Abstract

The special issue “Symbiotic Nitrogen Fixation in Legume Nodules: Metabolism and Regulatory Mechanisms” aims to investigate the physiological and biochemical advances in the symbiotic process with an emphasis on nodule establishment, development and functioning. The original research articles included in this issue provide important information regarding novel aspects of nodule metabolism and various regulatory pathways, which could have important future implications. This issue also included one review article that highlights the importance of using legume trees in the production of renewable biofuels.

Symbiotic N_2_ fixation is one of the biological processes important for development of sustainable agriculture by which the atmospheric N_2_ is converted to ammonia with the aid of a key enzyme called nitrogenase [1,2]. It is achieved by bacteria inside the cells of *de novo* formed organs, the nodules, which usually develop on roots of various leguminous plants. This process is resulted from the complex interaction between the host plant and rhizobia (used as a colloquial reference to *Rhizobium*, *Bradyrhizobium*, *Sinorhizobium* and *Mesorhizobium*). This mutualistic relationship is beneficial for both symbiotic partners; the host plant provides the rhizobia with carbon and source of energy for growth and functions while the rhizobia fix atmospheric N_2_ and provide the plant with a source of reduced nitrogen in the form of ammonium. Thus, the process offers an economically attractive and ecologically sound mean of reducing external inputs and improving internal resources [3]. To increase knowledge of this vital process of particular importance in sustainable agriculture, major emphasis should be laid on the nodule metabolism and various regulatory pathways which represent the central themes for the most conducted research in this area.

Legume nodules are very complex organs, containing several interacting processes that operate at distinct levels, including, at least, nodule formation, carbon metabolism, oxygen supply, cellular redox and transmembrane transport [1,2,3,4,5,6]. Nodule metabolism and regulation have been a topic of intensive research for quite a long time. Despite the enormous progress in this field, more research will still be required to provide greater understanding of this fantastic process [7,8,9,10,11]. An attempt has been made to provide up-to-date information that helps in fulfilling the gaps and giving answers for some pending questions. The present special issue of the *International Journal of Molecular Sciences* (*IJMS*) is entirely devoted to cover many new aspects in relation to the metabolic processes as well as the regulatory factor(s) which are intimately involved in the regulation of the symbiotic engine of various plants under normal and stressful conditions [12].

In this special issue, Janczarek and Rachwał discuss the possible role played by acidic exopolysaccharide (EPS) in the formation of efficient symbiosis between *Trifolium pretense* and *Rhizobium leguminosarum* [13]. The synthesis of EPS in rhizobia was shown to be regulated by several proteins at both transcriptional and post-transcriptional levels [14]. In their paper, the authors characterized a mutant strain having a Tn5 insertional mutation in *pssA* gene that is known to be critically involved in EPS synthesis. The results showed that certain pleiotropic effect in rhizobial cells was occurred, leading to several physiological and symbiotic defects which subsequently resulted in failure of host infection.

In the research article on *Medicago truncatula* by Cabeza *et al.* [15], the authors further extended previous studies, e.g., [16], by characterizing the systemic regulatory circuit known as AON (autoregulation of nodulation) from a carbon metabolic point of view. At optimal growth conditions, the supernodulating mutant (Mt*_sunn_*) showed that the photosynthetic supply was not the critical factor for the poor performance of the supernodulating mutant line. The authors concluded that the difficulties in controlling the activity of excessive number of nodules at whole plant level might be the reason for the low symbiotic efficiency in Mt*_sunn_* plants.

A research article by Saito and colleagues describe an experimental approach attempting to reveal new insights for the inhibition of soybean root nodules by nitrate supplementation [17]. With the aid of a digital camera, newly developed computer software and 2D-PAGE profiles of nodule proteome, the authors were able to report that nodule growth was rapidly depressed a few hours after 5 mM nitrate application. At the same time, the authors also investigated the effect of the 5 mM nitrate supply on the root system of nodulating plants. Interestingly, they reported that while the primary roots showed a similar response to nitrate supply as the nodules, *i.e.*, exhibiting growth retardation, the lateral roots displayed a reversible trend.

Delmotte *et al.* utilized the proteomics approach to study physiological responses of the photosynthetic *Bradyrhizobium* sp. ORS278, during its symbiotic association with the semi aquatical plant *Aeschynomene indica* that forms nodules on both roots and stems [18]. The paper published by this group demonstrated that the bacteroid proteomes of stem nodules and root nodules were highly conserved and the specific stem nodule proteins were related to the phototrophic ability of the investigated bacterium. Furthermore, the authors discovered that the *EtfAB* locus is particularly important for forming an efficient symbiotic association between *Aeschynomene*-*Bradyrhizobium*.

A research article by Dean and co-workers explore how strongly the modulation of *Bradyrhizobium japonicum* nodulation manipulated by soil nitrogen (N) supply could influence the interaction between soybean and herbivores of different feeding guilds [19]. In their investigation, the authors came across the fact that the mutualistic associations with rhizobia has an apparent effect on the plant nutritional quality and the induction of defense signaling pathways which collectively affect the herbivore feeding preferences and performance.

Zhang *et al.* [20] have extensively discussed recent research that was carried out to elucidate the molecular basis for the sensitivity of soybean to cold stress. The genome-wide expression analysis of miRNAs in response to chilling was particularly helpful as it revealed the miRNAs that are involved in response of mature nodules to cold stress. These findings have allowed the authors to conclude that miRNAs are involved in the protection against chilling injury in mature soybean nodules.

In addition to symbiosis’s substantial impact on sustainable food production and reducing the environmental and climatic impacts, the potential importance of the symbiotic relationship as an alternative source for sustainable production of biofuel feedstocks was discussed in the review by Biswas and Gresshoff [21]. In their review, the authors have also extensively analyzed the potential and discussed the benefits of legume trees as future energy crops, particularly in relation to their impact on nitrogen inputs and the net energy balance for biofuel production. According to the authors, legume trees are estimated to have minimal impact on global food supplies, land use, and commodity prices. The assessment of the criteria that determine the more relevant biofuel feedstock candidates places the prominent species *Pongamia pinnata* in a leading position for further consideration and utilization. Details on the process of *Pongamia* nodulation were reviewed in this article.

## Conclusions

We believe that the manuscripts published in this Special Issue could contribute to a better understanding of the legume symbiotic N_2_ fixation process. Several important and novel aspects of nodule metabolism and regulation were highlighted and discussed in this issue. The future implication of these findings could have an extremely positive impact on designing strategies to enhance legume productivity by genetic engineering. We hope that the readers will enjoy this Special Issue of *IJMS* and find useful and interesting information in this fascinating field of plant biology.
